# The Use of Machine Learning in Emergency Care Units: A Systematic Review

**DOI:** 10.1177/21501319251414821

**Published:** 2026-05-07

**Authors:** Jennifer Nkhwashu, Lizzy Ofusori, Patrick Ndayizigamiye, Tebogo Makaba

**Affiliations:** 1 Department of Applied Information Systems, University of Johannesburg, Johannesburg, South Africa; 2 Centre for Applied Data Science, University of Johannesburg, Johannesburg, South Africa

**Keywords:** artificial intelligence, emergency department, machine learning, patient assessment, systematic review, PRISMA, GRADE assessment

## Abstract

Emergency departments (EDs) are critical points of entry in healthcare systems where timely and accurate decision-making is vital. Machine learning (ML) offers promising capabilities to enhance patient triage, optimize resource allocation, and improve clinical outcomes in these high-pressure environments. This systematic review investigates the application of ML in EDs, identifies commonly used algorithms and tools, examines their limitations, and provides recommendations for improvement. A structured literature search was conducted across 5 major databases: Google Scholar, Scopus, Web of Science, IEEE Xplore, and PubMed, yielding 1257 peer-reviewed articles. Studies were included if they were published between 2017 and 2024, written in English, and focused on ML applications in EDs within the fields of Computer Science, Engineering, Decision Science, or Mathematics. Exclusion criteria eliminated articles under 6 pages, inaccessible full texts, non-ML-focused studies, and publications such as proposals, abstracts, or book reviews. After screening and quality assessment by 2 independent reviewers, 27 studies were selected for in-depth analysis. Of these, 88.9% were journal articles, 7.4% book chapters, and 3.7% conference proceedings. Findings reveal that the various ML algorithms applied in EDs are context-dependent and use various evaluation metrics, while tools for data extraction and analysis include Python, Keras, TensorFlow, SQL, MATLAB, RStudio, and IBM SPSS. The identified limitations involved data complexity, model accuracy, lack of generalizability, and incomplete datasets. Recommendations across studies emphasized the need to broaden data sources, integrate additional predictors, and improve algorithmic comparisons. This review contributes to the growing body of knowledge on ML in emergency care by synthesizing current practices, highlighting critical challenges, and offering practical directions for future research and implementation.

## Introduction

Emergency departments (EDs; also referred to as emergency care units [ECUs]) are created to provide health services to patients with critical conditions who seek medical attention. Recent studies have shown that as the demand for ED services continues to increase, insufficient resources and internal patient flow problems have resulted in many patients experiencing longer waiting times, which is commonly referred to as the boarding effect.^1^ This delay in assessment and treatment often leads to adverse health outcomes and dissatisfaction among patients and their families.^2^ As noted by Darraj et al^3^ one of the main challenges EDs face is overcrowding, which can cause extended wait times and delays in care, ultimately leading to negative outcomes for patients. The Centers for Disease Control and Prevention (CDC) reported that, in 2021, there were 43 ED visits for every 100 people.^4^ Over 50% of EDs in the United States reported overcrowding as a significant problem, with many reporting wait times exceeding 4 h.^4^ Overcrowding can also increase the risk of infection transmission and can have serious consequences for patient care, including delayed treatment and increased risk of medical errors.^5^ EDs also face challenges related to the shortage of healthcare professionals, which has put additional strain on EDs, leading to longer waiting times and reduced quality of care. In a survey conducted by the American Nurses Association (ANA), 71% of ED nurses reported that their department was understaffed.^6^ This shortage has led to increased stress and burnout among healthcare workers, negatively impacting patient care.^7^ Moreover, EDs face challenges related to social determinants and patient diversity in health. Social determinants of health, such as poverty and lack of access to health care, can impact patients’ health outcomes and increase the demand for ED services.^8^

According to Tschoellitsch et al,^9^ when a patient arrives at the ED, they are initially assessed based on the severity of their condition to prioritize those in need of immediate medical attention. This process, known as triage, involves evaluating the patient’s demographics, vital signs, and primary complaint to determine the urgency of treatment, particularly in emergency situations where many patients require admission.^10^

To address the healthcare needs of the large number of patients visiting EDs, Janke et al^11^ and Vela et al^12^ suggested the application of predictive analytics and machine learning (ML) to enhance admission rates as well as patient throughput and outcomes. This could lead to early identification of patients who require urgent medical attention and may enable better optimization of ED patient flow and improved utilization of resources.^13^ Historically, predictive analytics has played a role in risk categorization and diagnostics in clinical medicine.^14^ Moreover, with enhanced computing capabilities now accessible to clinicians, predictive models are used to leverage advanced techniques such as ML to assess risks.^11^ Additionally, the systematic collection of patient data from prospective registries and health information exchanges is considered to define both the predictor and the outcome variables for a new predictive model. The effectiveness of these predictive models depends on the accuracy and timeliness of clinical interactions, with triage information playing a crucial role in improving the accuracy of admission predictions.^11^

Several factors may account for the gradual increase in the use of ML in the context of ED operations. ML approaches can incorporate high-order nonlinear interactions between predictors, which cannot be addressed by traditional modeling approaches (eg, logistic regression).^15^ The use of advanced ML algorithms allows for the evaluation of far more clinical variables than in traditional modeling approaches, with the added benefit of discovering clinical variables not expected to be of predictive value or which otherwise would have been omitted as a rare predictor.^16^ ML has attracted attention due to its ability to process and provide complex nonlinear relationships and a more stable forecast. However, despite the growing demand for ML in health care, there are limited studies on the practical applications of these techniques in EDs. Hence, this study aimed to provide insights into the application of ML in EDs. It sought to present information on the range of ML algorithms used in EDs, their limitations, and recommendations to address the limitations. Consequently, this systematic review was anchored on the following 4 research questions (RQs) related to the application of ML in the EDs:

**RQ1:** Which ML algorithms are used in EDs?
**RQ2:** What are the ML tools used for data extraction, analysis, and optimization?
**RQ3:** What are the limitations of ML algorithms used in EDs?
**RQ4:** What are the recommendations to address the limitations?

The remainder of the paper is structured as follows: Section 2 outlines the research methodology employed in the study. Section 3 discusses the findings in the context of the research questions, contributing to the understanding of ML applications in EDs. Section 4 highlights the study’s limitations and suggests directions for future research, and Section 5 details the study’s contributions.

## Methods

A systematic literature review (SLR) was used to investigate the range of ML capabilities that are used in EDs and their limitations. An SLR is a structured and comprehensive method of reviewing and synthesizing existing research on a specific topic or question. Unlike traditional narrative reviews, an SLR follows a rigorous and transparent process to identify, evaluate, and summarize all relevant studies, minimizing bias, and ensuring reproducibility.^17^ Therefore, this method was chosen to gather and summarize current evidence on various ML techniques used in EDs and their limitations, which were analyzed to inform how the healthcare sector can leverage their benefits. The SLR was guided by the Preferred Reporting Items for Systematic Reviews and Meta-Analyses (PRISMA) guidelines to ensure methodological rigor and transparency. PRISMA is a widely recognized framework for conducting and reporting systematic reviews.^18^ It provides a systematic, structured method to identify and select appropriate studies while maintaining clarity and reproducibility.

### Search Strategy

This study’s data were obtained from Google Scholar, Scopus, Web of Science (WoS), IEEE Explorer, and PubMed databases. According to García-Peñalvo,^19^ integrating several databases increases the depth and accuracy of literature reviews. In addition, using several databases provides a broader coverage, which helps map out smaller research areas.^20^ The broader coverage is significant in the context of the use of ML in EDs. This is because different academic databases serve specific purposes and focus areas, meaning they often include unique journals, conference papers, or other materials that may not be available in other databases.^20^ This lack of overlap is why searching multiple databases is crucial when conducting an SLR, especially in an interdisciplinary field such as using ML in EDs. Articles identified through the database search were evaluated for eligibility using the following primary search string:

*((“machine learning” OR “deep learning”) AND (“emergency care” OR “emergency department” OR “emergency unit” OR “emergency care” OR “trauma care”)).*

These databases were accessed on November 19, 2024.

### Inclusion and Exclusion Criteria

The inclusion and exclusion criteria are presented in Table 1. The subject area of the search string was limited to the disciplines “Computer Science,” “Engineering,” “Decision Science,” and “Mathematics.” In addition to using the search string to identify articles, additional criteria were subsequently applied to refine the search results and include only records that satisfied the following conditions: (i) authored in English only; (ii) of all types, excluding Correction, Abstract, Book Review, Data Paper, Lecture Notes, Letter, and Proposal, and (iii) publications that employ predictive analytics, ML, and deep learning (DL) in EDs. The duration period for the literature search was not explicitly defined to ensure a comprehensive and unbiased collection of relevant studies. However, after searching, it became evident that most articles addressing the use of ML in EDs were published between 2017 and 2024. This period represents a surge in research interest due to advancements in ML techniques and their increasing applicability in healthcare settings. By narrowing the focus to this timeframe, the review captures the most current and relevant developments in the field, ensuring the findings reflect contemporary practices and innovations.

**Table 1.** Inclusion and Exclusion Criteria.

S/no.	Inclusion	Exclusion
1.	Articles published between 2017 and 2024	Publication that is a doctoral symposium
2.	The study is written in English	The full text of the publication is not available
3.	The focus of the publication is on using ML in an ECU	Research that does not employ predictive analytics, ML, and DL in EDs
4.	Subject areas: Computer Science, Engineering, Decision Science, and Mathematics	Duplicated articles are excluded
5.	The study is peer-reviewed	The paper is less than 6 pages in length
6.	The full text of the study is available	The publication includes corrections, abstracts, data papers, lecture notes, book reviews, letters, and proposals

### Selection Process

**Figure 1.**
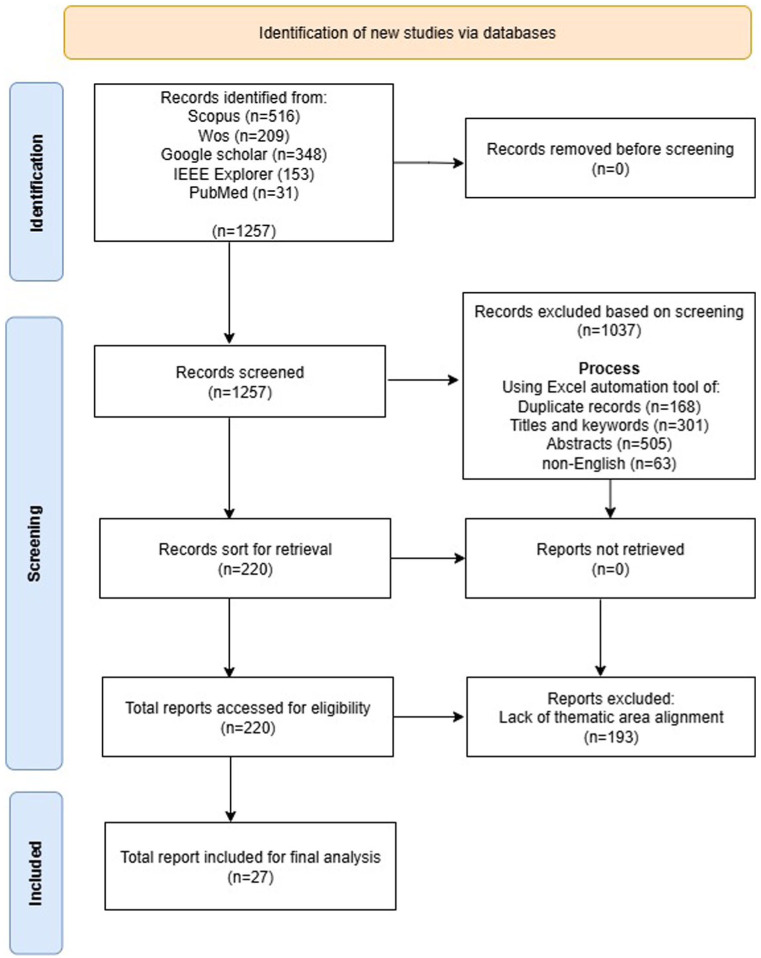
PRISMA flow diagram for the use of ML in ED.

As illustrated in Figure 1, the PRISMA framework comprises a checklist and a flow diagram designed to promote transparent and comprehensive reporting of research.^21^ The PRISMA standard includes screening, identification, eligibility, and inclusion.^18^ During the identification phase, the search string described in Section 2.1 was applied to retrieve articles from the 5 databases. In the screening phase, an Excel worksheet was used as an automation tool to list all extracted papers with the corresponding databases from which they were retrieved. Duplicate articles were then located across the 5 databases and removed. Subsequently, each article’s title and abstract were scrutinized to ensure their relevance to the application of predictive models in EDs. If the paper’s abstract was not in line with the use of predictive models in EDs, it was not included in the study. In the eligibility phase, the full paper was read to ensure its relevance to the context of the study, that is, to ensure the paper qualified to answer any of the research questions that underpinned this systematic review. A total of 1257 articles were identified from the 5 databases. Through the automation tool (Excel), 168 duplicate papers were removed, 301 were excluded based on title and keyword criteria, and 505 were excluded based on abstracts alone. In addition, 63 non-English papers were excluded, and 193 papers that did not align with the thematic area were removed. Ultimately, 27 papers were successfully retrieved and included in the final analysis.

For the quality assessment, 2 independent researchers evaluated the selected papers to ensure their relevance and quality for this study. This evaluation considered factors such as research objectives, prior studies, and literature indexes. Index identification was performed by referencing Scimago Journal and Country Rankings. An assessment scoring system ranging from 0 to 1, based on the criteria outlined in Table 2, was applied. A score of 0 indicates that the study does not meet the specified checklist criteria, a score of 0.5 indicates that the criteria are implicitly defined, and a score of 1 indicates strong alignment with the criteria. Based on the checklist criteria adapted from other studies, a minimum of 5 out of 8 scores was needed for a paper to be selected for final consideration.^22^ This minimum requirement was essential to ensure that the checklist standards were followed for each paper.^23^ The reliability and validity of the study findings are enhanced by the scoring system, because it ensures the integrity of the research outcomes.^24^ Table 3 depicts the score results of each paper that was evaluated. Ultimately, 27 papers were selected based on this assessment process.

**Table 2.** Quality Assessment Criteria.

Checklist	Question checklist
C1	Is the purpose of the research explicitly stated in the article?
C2	Is there related work from previous research to show the study’s main contribution?
C3	Is there a background stating the research context in the study?
C4	Is there a literature review in the study?
C5	Is there a conclusion that is relevant to the purpose of the research?
C6	Are there outcomes from the article?
C7	Are there recommendations for future work that fit the SLR context?
C8	Are the articles Scimagojr rated (Q1/Q2/Q3/Q4)?

**Table 3.** Quality Assessment Results.

Author	Criteria								Total
	1	2	3	4	5	6	7	8	
25	1	1	1	1	1	1	1	1	8
26	1	1	1	1	1	1	1	1	8
27	1	1	1	1	1	1	1	1	8
28	1	1	1	1	1	1	1	1	8
29	1	1	1	1	1	1	1	1	8
30	1	1	1	1	1	1	1	0	7
31	1	1	1	1	1	1	1	1	8
15	1	1	1	1	0.5	1	1	0	6.5
32	1	1	1	1	1	1	1	1	8
33	1	1	1	1	1	1	1	1	8
34	1	1	1	1	1	1	1	0	7
35	1	1	1	1	1	1	1	1	8
36	1	1	1	1	1	1	1	1	8
37	1	1	1	1	1	1	1	0	7
38	1	1	1	1	1	1	1	0	7
39	1	1	1	1	1	1	1	0	7
40	1	1	1	1	1	1	1	1	8
41	1	1	1	1	1	1	1	1	8
42	1	1	1	1	0.5	1	1	0	6.5
43	1	1	1	1	1	1	1	0	7
44	1	1	1	1	1	1	1	1	8
45	1	1	1	1	1	1	1	0	7
46	1	1	1	1	1	1	1	0	7
47	1	1	1	1	1	1	1	0	7
48	1	1	1	1	1	1	1	1	8
49	1	1	1	1	1	1	1	0	7
50	1	1	1	1	1	1	1	1	8

### Data Extraction and Synthesis

Data extraction from the selected research papers was carried out by 2 independent reviewers using the following structured criteria:

i. Does the paper identify the ML algorithms used in EDs?
ii. Does the paper examine the ML tools applied for data extraction, analysis, and optimization?
iii. Does the paper discuss the limitations of ML algorithms used in EDs?
iv. Does the paper offer recommendations to address these limitations?

The extracted papers from both researchers were compared, and any discrepancies were resolved through mutual agreement. As noted by Mallett et al,^51^ discrepancies in studies of this nature can be minimized when researchers collaboratively review and align their coding decisions to ensure consistency and relevance. Following reconciliation, the papers were synthesized based on key thematic categories. This thematic synthesis was instrumental in providing a structured understanding of the application of ML in EDs.

### Certainty of Evidence Assessment (GRADE Framework)

The study employed the GRADE (Grading of Recommendations, Assessment, Development, and Evaluation) approach, as described by Guyatt et al,^52^ to assess the certainty of evidence presented in the 27 studies. The GRADE framework rates the certainty of evidence across 5 domains, namely: (i) risk of bias, (ii) inconsistency, (iii) indirectness, (iv) imprecision, and (v) publication bias. The study adapted the standard GRADE guidance for prognosis and prediction-model studies, focusing on the performance and validation of emergency care ML tools rather than treatment effects.^53-55^ Observational studies, which form the basis of almost all prognostic and prediction-model research, start at “low” certainty in GRADE and may be rated down by 1 or 2 levels for serious or very serious concerns in any of the 5 domains; in some circumstances, they may be rated up when the evidence is particularly strong, for example, when studies have used very large, consistent, and directly applicable datasets.^56^ For each study, the 5 domains for certainty of evidence were assessed as follows:

-  Risk of bias: Based on design (retrospective vs prospective), clarity and completeness of reporting, handling of missing data, risk of overfitting, and whether model performance was assessed in an independent sample. Single-center development studies that relied solely on internal resampling and lacked transparent reporting were rated as having a serious risk of bias, particularly for complex DL models that lacked clear handling of overfitting or missing data. Large, well-reported, multi-site or national datasets with clear model-building, and validation procedures were judged as not having a serious risk of bias.
-  Inconsistency: As most ML studies provided a single development-validation dataset rather than replication across multiple independent cohorts, the study focused on internal inconsistency in performance, for example, large variation across outcomes or subgroups, and, when relevant, performance differences across external validation sites. Most studies did not present conflicting effect estimates, so the inconsistency was assessed to be not serious.
-  Indirectness: Examined how closely the study population, setting, predictors, and outcomes matched the context of the study. Studies conducted solely in pediatric EDs, intensive care units (ICUs), or highly specialized contexts, or those using administrative data such as operational metrics, for example, length of stay, rather than frontline ED decision outcomes, were typically downgraded for serious indirectness. Conversely, indirectness was deemed not serious in national ED datasets, multi-hospital cohorts, and models targeting direct ED decisions such as triage priority, admission, critical care, and early mortality.
-  Imprecision: Examined the total sample size, number of outcome events, confidence intervals around discrimination or calibration metrics, and the complexity of the outcome structure. Small single-center studies with limited events, wide or unreported confidence intervals, and complex multi-class outcomes were downgraded for serious imprecision. Large cohorts, particularly national or multi-center datasets with narrow confidence intervals and stable discrimination estimates across subgroups, were deemed as not serious for imprecision.
-  Publication bias: Systematic detection of publication bias is challenging in prediction-model research. In line with GRADE guidance for prognosis and model studies, we looked for strong asymmetry in study results, selective reporting of favorable metrics, or evidence of multiple small underperforming studies being absent.^54,57^ No direct evidence of substantial publication bias emerged, so this domain was rated as “undetected” for all studies.

For each study, these domain-level judgements were combined into an overall GRADE rating (high, moderate, low, or very low). Observational ML studies started at low certainty. They were then downgraded 1 level (to very low) when at least 1 domain showed serious limitations, or when multiple domains showed borderline concerns. They were downgraded 2 levels (from low to very low) when 2 or more domains showed clearly serious problems, for example, high risk of bias and serious indirectness, and imprecision in small, single-center models. Studies were upgraded from low to moderate or high when they included very large, representative datasets (national or multi-country ED populations), direct outcomes, and strong, consistent model performance, often with external validation and calibration assessment, in line with adapted GRADE guidance for prognostic and prediction model evidence.^53,55,56^ The results of the GRADE assessment are described in Categorization of Areas of Investigation Section.

## Results

This section presents a descriptive analysis of the papers extracted through the PRISMA process. It then provides an analysis of the retrieved papers based on the guiding research questions.

### Description of the Papers Based on the Year of Publication and Document Type

The annual publication count is presented in Table 4. The table provides a summary of research papers published between 2017 and 2024, grouped by the year of publication, key author(s), and the paper count. In 2017, only 1 article^25^ was published, accounting for 3.7% of the total articles reviewed. This reflects a relatively early contribution to the body of knowledge under review. In 2018, a significant increase in research activity is evident, with 6 articles^26-31^ published, accounting for 22.2% of the total reviewed articles. This marks a notable growth in interest and research output in the field. In 2019, the number of publications slightly increased to 7,^15,32-37^ which corresponds to 25.9% of the total articles reviewed. This year ties with 2020 for the highest number of articles, indicating a peak in research activity. In 2021, 2022, and 2023, research output dropped significantly, each producing only 1 paper, contributing just 3.7% per year. This may indicate a period of consolidation or reduced research activity on the topic. A modest increase was observed in 2024, with 3 papers comprising 11.1% of the total reviewed papers. This trend indicates a sharp rise in publications during 2019 to 2020, followed by a decline and a slight resurgence in 2024.

**Table 4.** Distribution of Reviewed Articles Included in the Study by Year of Publication.

Year of publication	Authors	Paper count	
		Number of papers	Percentage count
2017	25	1	3.7
2018	26, 27, 28, 29, 30, 31	6	22.2
2019	15, 32, 33, 34, 35, 36, 37	7	25.9
2020	38, 39, 40, 41, 42, 43, 44	7	25.9
2021	45	1	3.7
2022	46	1	3.7
2023	47	1	3.7
2024	48, 49, 50	3	11.1

The 27 retrieved papers were then categorized into 3 major document types. Most records (88.9%, N = 24) were published as journal articles, followed by book chapters (7.4%, N = 2), and conference proceedings (3.7%, N = 1).

### Categorization of Areas of Investigation

Across the 27 included studies, 4 recurring areas of investigation were identified (see Table 5). Fifteen studies^15,25,26,28,29,33,35,36,38-41,44,45,47^ focused on ED triage and early-visit risk stratification (Category A), where models use information available at or shortly after triage, such as vital signs, triage category, chief complaint, and, in some cases, triage notes, to predict critical illness, hospital admission, or early adverse outcomes. A second group, consisting of 5 studies,^32,34,37,42,49^ targeted ED operational outcomes (Category B), such as forecasting the number of emergency admissions or predicting prolonged ED length of stay, to support bed management and flow. Four studies^27,30,31,48^ utilized longitudinal primary-care and hospital data to estimate the population-level risk of future emergency admissions (Category C), informing proactive case management and risk stratification at the system level. Finally, 3 studies^43,46,50^ addressed post-admission in-hospital or ICU outcomes (Category D), including in-ICU mortality among patients with ICU-acquired infections, reflecting downstream risks that remain highly relevant to emergency care planning.

**Table 5.** ML and DL Algorithms Used by the Authors.

S/no.	Study design	Categorization	ML and DL algorithms	Evaluation metrics	Data source	Sample size	Summary of the findings	Authors
1	Retrospective, cross-sectional study	A	RF	Emergency Severity Index (ESI)	Single United States health system EHR: 1 urban academic ED and 1 community ED	172 726 ED records	E-triage showed strong accuracy (AUC 0.73-0.92) and matched or outperformed ESI in identifying clinical patient outcomes.	26
2.	Retrospective study	B		AUROC, specificity, sensitivity, and accuracy	Administrative data from the German system of diagnosis-related groups (DRGs)	7481 records of patients who required emergency care from the German system between 2005 and 2013	The study accurately (96%) classified and categorized inpatient admissions as either emergency or elective care, achieving a very high AUROC curve (>0.99).	32
3	Prospective cohort study	A	GBT, SVM, RF, and LR	AUC, accuracy, sensitivity, and specificity	Multicenter Canadian interRAI ED study: 8 EDs across 5 provinces: Nova Scotia, Ontario, British Columbia, Manitoba, and Saskatchewan	Data included 6847 visits by 2274 older adults (≥75 years) between November 2009 and April 2012	GBTs achieved the highest accuracy (AUC = 0.80) for predicting hospitalizations among older ED patients, with the most influential predictors being home intravenous therapy, time of ED arrival, need for formal support, walking independence, and presence of an unstable medical condition.	38
4	Retrospective cohort study	A	GBM	AUC, sensitivity, specificity, negative predictive value (NPV), positive predictive value (PPV), and false-positive rate (FPR)	Data of consecutive adult patients admitted to the academic ED of 1 tertiary center were retrieved from the hospital’s EMRs	Data of consecutive adult patients (ages 18-100) admitted to the ED of 1 hospital were retrieved (January 1, 2012-December 31, 2018)	The ML model accurately predicted early and short-term mortality among ED patients using routinely collected triage-level data, achieving an AUC of up to 0.962.	39
5	Retrospective cohort study	A	XGBoost, SVM, and DNN	AUC, sensitivity, specificity, accuracy, PPV, and NPV	Data from a pediatric hospital located in São Paulo, Brazil	The study used 499 853 ED presentations of patients up to 18 years reserved over 3.5 years (between January 2015 to August 2018)	A DL-enhanced ML model combining structured and textual triage data accurately predicted pediatric ED admissions (AUC = 0.892), with text features significantly improving performance beyond traditional triage systems.	40
6	Retrospective study	A	CNN	AUROC and accuracy	National Hospital Ambulatory Medical Care Survey (an open dataset) was used	118 602 patient visits of United States EDs from 2012 to 2016	A CNN model using data-to-image transformation accurately predicted ED hospitalizations using national survey data, achieving an AUROC of 0.86 and showing strong potential to support more objective triage decision-making.	33
7	Retrospective study	C	XGBoost and RNN	AUC and accuracy	Real-world hospital EHR visit logs; source institution not reported.	Data extracted from original data that contained 6000 patients	Non-linear ML models (XGBoost and RNN) outperformed traditional linear approaches in predicting whether patients will visit the emergency room and in estimating ER visit counts, with sequence-based features further improving prediction accuracy.	27
8	Retrospective cohort study	A	LR, DT, and GBM	AUC, sensitivity, specificity, precision, or PPV	Data collected from 2 major acute hospitals in Northern Ireland	Administrative data: 120 600 records	GBM outperformed LR and DT in predicting ED admissions, showing potential for supporting real-time resource planning and flow management.	28
9	Retrospective cohort study	A	RF, GBDT, DNN, and LR	AUC, accuracy, sensitivity, specificity, PPV, and NPV	Netherlands Department Evaluation Database (NEED)	Sample data collected from 3 hospitals using the NEED between 1 January 2017 and 31 December 2019	GBDT model performed best over time with an AUC of 0.84 (0.77-0.88) at triage, 0.86 (0.82-0.89) at 30 min, and 0.86 (0.74-0.93) after 2 h.	45
10	Retrospective cohort study	A	LR, XGBoost, and DNN	AUC and accuracy	Single US health system EHR (Yale New Haven Health): 1 academic and 2 community EDs	972 patients (March 2014-July 2017)	ML and LR models using triage data, patient history, or both, accurately predicted hospital admission at ED triage, achieving AUCs up to 0.92 with full-variable models performing best and requiring only half the available training data to reach optimal performance.	29
11	Retrospective study	B	LR, RF, GBDT, XGBoost, and an ensemble model of the 4 models	AUC	Data from the Nephrology Department of the ASC of West China Hospital	Not specified	XGBoost accurately prioritized elective patient admissions using historical registration data, showing potential to automate and improve the hospital admission screening process.	34
12	Retrospective cohort study	C	GTB, RF, and SVM	AUROC, sensitivity, specificity, and accuracy	Data from patients undergoing radiotherapy (RT) or chemoradiotherapy (CRT)	A total of 8134 outpatient courses of RT and CRT from a single institution from 2013 to 2016 were identified	ML models, especially GBT (AUROC: 0.798), accurately predicted emergency visits and hospitalizations during radiotherapy or chemoradiotherapy, with extensive EHR data significantly improving performance and highlighting key pretreatment clinical predictors.	30
13	Retrospective longitudinal cohort study	C	RF and GBC	AUC	Data from the linked EHR from the UK Clinical Practice Research Datalink (CPRD)	4.6 million patients aged 18 to 100 years from 389 practices between January 1985 and September 2015 across England	Gradient boosting (GBoost) substantially outperformed Cox regression for predicting first emergency admissions, achieving an internally validated AUC of 0.848 and externally validated AUC of 0.826 (vs 0.740 and 0.788 for Cox regression), with temporal variable enrichment further improving discrimination and calibration across all predictions.	31
14	Retrospective cohort study	A	LR, ANN, DT, RFSVM, and extreme gradient	AUC, accuracy, sensitivity, and specificity	Data from a metropolitan area teaching hospital in the United States	Approximately 50 000 ED visits per year. The study presents data collected from 2006 to 2009	XGBoost achieved the highest predictive performance for ED admission, with AUC improving from 0.83 to 0.86 as data volume increased, outperforming models such as LR, DT, RF, NN, and SVMs (which showed decreasing AUC), showing its suitability for efficient and accurate hospital admission prediction.	35
15	Retrospective cohort study	A	LR, RFs, and a random undersampling boosting algorithm	AUROC, specificity, PPV, true negative rate (TNR), and accuracy	Emergency Department Information Systems (EDIS) of a hospital in Portugal and a hospital in the United States	A total of 599 276 and 267 257 ED patient visits. The data extracted range from 2012 to 2016	LR models using triage vitals and natural language-processed chief complaints accurately identified ICU-risk patients, achieving AUCs of 0.91 (PR-AUC 0.30) in the United States hospital and 0.85 (PR-AUC 0.06) in the hospital in Portugal. These models substantially improved recall for under-triaged MTS/ESI-3 patients compared to standard triage-only models.	41
16	Retrospective cross-sectional study	A	LR and NN	AUC	National Hospital Ambulatory Medical Care Survey (NHAMCS)	A sample of 54 320 ED visits from approximately 300 hospitals in the United States from 2012 to 2013	LR and NN models predicted hospital admission or transfer with good accuracy, reaching AUCs of 0.846 (LR) and 0.844 (Multi-layer Neural Network-MLNN) when structured triage data were combined with NLP-derived free-text features. The findings further show that adding patient chief-complaint text improves predictive performance regardless of the modeling approach.	25
17	Retrospective cohort study	B	LR, SVM, and a feedforward neural network	AUROC and accuracy	EHR data from Oxford University Hospitals (OUH) were collected	9324 patients’ records from EDs spanning January 2013 to April 2017 were considered	A DL model using ED triage data successfully predicted patients’ hospital admission location across 7 ward types with AUROC values ranging from 0.60 to 0.78 and an overall accuracy of 52% (vs 14% by chance), showing potential to support hospital bed planning and resource allocation.	42
18	Retrospective cohort study	D	LR, RF, SVM, and NN	AUC and Brier Score	Data from the Korea National Hospital Discharge Survey (KNHDS)	“8937 samples covering the period from 2008 to 2017, categorized as drug intoxication (ICD-10 codes T36.0-T65.9)”	LR in-hospital mortality after emergency admission with strong performance, calibration slope of 0.90, and AUC of 0.847, performing competitively with more complex ML methods and showing no evidence of overfitting.	43
19	Retrospective cross-sectional study	A	LR, RF, GB, DT, DNN, and Lasso regression	AUC, sensitivity, specificity, PPV, and NPV	United States National Hospital and Ambulatory Medical Care Survey (NHAMCS)	All adult patients (aged ≥18 years). ED data from 2007 through 2015 were utilized	ML models significantly outperformed conventional ESI-based LR in predicting critical care (AUC 0.86 vs 0.74) and hospitalization (AUC 0.82 vs 0.69) outcomes, yielding a greater net clinical benefit and reducing under- and over-triage across severity levels.	36
20	Retrospective cohort study	D	LR, RF, SVM, XGBoost, LightGBM, and MLP	AUC, accuracy, sensitivity, specificity, NVP, and PPV	Data from ED patients from 3 hospitals (Linkou Chang Gung Memorial Hospital, Kaohsiung Chang Gung Memorial Hospital, and Keelung Chang Gung Memorial Hospital)	52 626 adult ED patients with pneumonia	ML models showed strong predictive performance, with RF achieving an AUC of 0.781 for sepsis/septic shock, and LightGBM achieving AUCs of 0.847 for respiratory failure and 0.835 for mortality. The LightGBM-based AIoT system was implemented clinically and demonstrated lower sepsis/septic shock rates compared with usual care.	46
21	Retrospective study	A	LR, DNN, and GTB	AUC, sensitivity, specificity, PPV, and NPV	Single urban academic tertiary-care ED in northeastern United States; de-identified triage EHR and chief-complaint text	445 925 patients from January 1, 2012, to January 1, 2020, were included	DNN incorporating chief-complaint text accurately identified critically ill ED patients, achieving an AUC of 0.851, outperforming vital-sign thresholds (0.521), ESI (0.672), LR (0.803), a 2-layer NN (0.811), and GBoost (0.820).	44
22	Retrospective study	B	LSTM	Mean square error (MSE), Root mean square error (RMSE), Mean absolute error (MAE), Coefficient of determination (*R*^2^), and Mean absolute percentage error (MAPE)	Data from the pediatric ED at Lille Regional Hospital Center, France	24 000 patients over 2 years (2011 and 2012)	Using 2 years of historical pediatric ED attendance data, the LSTM model demonstrated excellent forecasting performance, achieving *R*² = .972, RMSE = 0.089, MAE = 0.068, and a mean forecast error of 5 patients per day, showing strong capability for predicting daily ED admissions.	37
23	Retrospective cohort study	A	LR, DT, KNN, SVM, MLP, GBDT, XGBoost, AdaBoost, and RF	Accuracy, Precision, Recall, and F1-score.	Dataset from a single-center ED of a university hospital in Istanbul with an annual visit of 200 000 patients	Data were collected from 2688 patients who visited the ED between April 1, 2020, and June 9, 2020.	RF performed best, achieving 89.1% micro accuracy, 89.0% precision, 89.1% recall, and 89.0% F1-score in distinguishing critical outcomes (mortality/ICU) from less critical ones (discharge/hospitalization), highlighting the strong potential of ML to augment triage.	47
24	Retrospective prognostic study	A	Lasso regression, RF, GBDT, and DNN	Sensitivity, specificity, PPV, NPV, positive likelihood ratio (PLR), and negative likelihood ratio (NLR)	Data from the National Hospital Ambulatory Medical Care Survey (NHAMCS)	A sample of 52 037 children’s health records, covering the period from January 1, 2007, to December 31, 2015, was collected.	DNN improved the prediction of critical care (*C*-statistic = 0.85 vs 0.78 for conventional triage) and hospitalization (*C*-statistic = 0.80 vs 0.73, *P* < 001), reducing under-triage of critically ill children and over-triage of less ill children across triage levels.	15
25	Retrospective cohort study	C	ANN, RF, GLM, NB, and XGBoost	Sensitivity, AUROC, and area-under-precision-recall-curve (AUPRC)	Data from EHRs of approximately 4.8 million Scottish residents (2013-2018)	4.8 million Scottish residents (2013-2018)	The study developed the SPARRAv4 ensemble ML score to predict 1-year emergency admission or death, achieving an AUROC of about 0.80 and demonstrating improved discrimination and calibration over previous Scottish risk scores with stable performance over a 3-year horizon.	48
26	Retrospective cohort study	B	RF, NN, LR, NB, and NN based on a multilayer perceptron (MLP)	Accuracy, precision, and recall	Data from San Giovanni di Dio e Ruggi d’Aragona University Hospital, Italy.	Dataset consisted of 496 172 admissions from 2014 to 2019	The RF model performed best, achieving 74.8% accuracy, 72.8% precision, and 74.8% recall, indicating that basic demographic and workflow variables can meaningfully predict ED-LOS and support crowding management.	49
27	Prospective cohort study	D	RF, GBM, and LR	AUROC, sensitivity, and specificity,	International Nosocomial Infection Control Consortium (INICC)-based surveillance database from a single tertiary hospital (Nemazee Hospital, Shiraz, Iran)	968 patients in 9 adult ICUs from February 2014 to June 2021	RF and GBM both achieved AUROC 0.77 (vs 0.74 for logistic regression), with sensitivities/specificities of 0.65/0.77 (RF), 0.79/0.62 (GBM), and 0.74/0.67 (LR) and Brier scores of 0.111, 0.105, and 0.114, respectively, with GBM showing the best overall performance for predicting risk of in-ICU death and potential to guide targeted interventions.	50

### GRADE Certainty of the Evidence Assessment

Across the 27 studies, overall certainty ranged from very low to high. Five studies were rated as very low^15,25,34,37,40^ certainty, 8 as low,^27,28,30,35,42,47,49,50^ 5 as moderate,^26,29,32,38,39^ and 9 as high^31,33,36,41,43-46,48^ (see Appendix A). High-certainty evidence came from large national or multicenter ED cohort studies that evaluated ML models on direct ED outcomes, typically using multi-region or national datasets and including both external validation and calibration assessment. These studies demonstrated a low risk of bias, direct applicability to ED practice, precise estimates, and no evidence of important inconsistency, aligning with the GRADE criteria for high certainty in observational evidence when data are large, consistent, and directly applicable.^56,58^

Moderate-certainty ratings were assigned to studies that used large multi-site or national datasets but lacked fully independent external validation or had modest concerns in 1 domain (typically imprecision or indirectness). Examples include multi-center geriatric admission models and large single-system cohorts where temporal validation was used but cross-system validation was absent.^39^ In these studies, the body of evidence remained fairly strong, yet showed concerns about generalizability or precision, which prevented them from being upgraded to high certainty.

Low- and very-low-certainty evidence was concentrated among single-center and context-restricted ML studies, and studies focused on operational outcomes such as length-of-stay, rather than core ED decisions. These studies frequently required downgrades for indirectness (limited match to broad ED populations and decisions), imprecision (small samples, few events, and wide or unreported confidence intervals), and, in some cases, risk of bias (internal-only validation with limited reporting of model development and calibration). Such patterns are consistent with GRADE applications in prognostic research, where indirect populations, small or unstable event counts, and single-center designs commonly drive downgrades in certainty.^53-55^

None of the 27 studies showed clear inconsistency or direct evidence of publication bias, largely because most ML articles in this field report a single model in 1 dataset, and comparative meta-analysis or funnel-plot type assessment is rarely feasible. Instead, most downgrades were driven by risk of bias, indirectness, and imprecision, while upgrades to moderate or high certainty were reserved for studies meeting emerging GRADE guidance for strong prognostic and prediction-model evidence: large, representative ED populations, clinically central outcomes, strong and stable model performance, and the presence of external validation and calibration reporting. Appendix A provides a concise explanation of the grading approach across the 5 domains.

### ML Algorithms Used in EDs (**RQ1**)

The first research question (**RQ1**) examined the various ML algorithms applied to build ML models in the context of ECUs. It is important to note that some papers also include DL algorithms. Table 5 lists the ML and DL algorithms used by the authors in the selected and reviewed publications.

*Random Forest (RF)*: Levin et al^26^ study assessed an electronic triage system (e-triage) utilizing ML to predict acute outcomes and enhance patient differentiation. Conducted as a multi-site, retrospective, cross-sectional study, it analyzed 172 726 ED visits across urban and community EDs. The e-triage system employs an RF model, leveraging triage data such as active medical history, chief complaints, and vital signs to simultaneously predict the need for inpatient hospitalization, critical care, and emergency procedures. These predictions are converted into triage-level designations. The study compared e-triage performance against the Emergency Severity Index (ESI), evaluating primary outcomes and secondary measures such as elevated troponin and lactate levels. Similarly, Krämer et al^32^ developed a model to classify inpatient admissions as either emergency or elective care, assigning a numerical urgency value. Using supervised ML techniques (specifically RF), the model was trained on physician-expert judgments, achieving 96% accuracy and an area under the Receiver Operating Characteristic (ROC) curve exceeding 0.99. This study offers the first comprehensive classification and urgency categorization for inpatient emergency and elective care, mapping urgency values to all relevant diagnoses in the International Classification of Diseases (ICD) catalog. The model integrates seamlessly with existing hospital data systems.

*RF, Gradient Boosting Trees (GBT), Support Vector Machine (SVM), and Logistic Regression (LR)*: Mowbray et al^38^ utilized various ML algorithms to predict ED admission among older adults, exploring clinical and policy implications. Their study analyzed data from the interRAI multinational ED study, focusing on 2274 Canadian ED patients aged 75 years and older, collected from 8 ED sites between November 2009 and April 2012. Predictors, drawn from the interRAI ED Contact Assessment, included geriatric syndromes, functional assessments, and baseline care needs. The study reported accuracy, sensitivity, and specificity for each model to enhance performance interpretation.

*Gradient Boosting Machines (GBM)*: Klug et al^39^ assessed a cutting-edge ML model, specifically GBM, to predict mortality at the triage level, aiming to improve patient categorization in the ED. They analyzed data from consecutive adult patients (aged 18-100 years) admitted to a single hospital’s ED between January 1, 2012, and December 31, 2018, validating the model’s effectiveness as an automated triage tool.

*Extreme Gradient Boosting (XGBoost), SVM, and Deep Neural Network (DNN)*: Roquette et al^40^ study proposed and compared predictive models for hospital admission using both structured and unstructured data available at triage. The dataset included 499 853 pediatric ED visits (admission rate of 5.76%) for patients 18 years and younger, collected over 3.5 years. Their optimal model employed a 2-stage architecture: a DNN to process textual data, followed by a gradient boosting classifier (GBC). This model achieved an Area Under the Curve (AUC) of 0.892 on test data. The study highlights the value of DNN-based text processing, as excluding text features reduced the AUC by approximately 2 percentage points.

*Convolutional Neural Network (CNN)*: Yoo et al^33^ introduced a system leveraging patients’ ED electronic health records (EHRs) to predict hospitalizations following completed ED procedures. Unlike most related studies, which rely on traditional ML for triage-related classification and emphasize feature selection, their approach transforms data into images and employs a CNN as the classifier. The system was validated using an open dataset from the National Hospital Ambulatory Medical Care Survey, encompassing 118 602 ED patient visits in the United States from 2012 to 2016. The model achieved an Area Under the Receiver Operating Characteristic Curve (AUROC) of 0.86 and an accuracy of 0.77.

*XGBoost and Recurrent Neural Network (RNN)*: Qiao et al^27^ developed predictive models that could forecast future emergency room (ER) visits by using 2 non-linear models, namely, XGBoost and RNN. The study utilized large-scale EHR data from a healthcare system, including variables such as demographic information, diagnosis codes, medication prescriptions, laboratory test results, and prior ER visits. Experimental results showed that both methods had better performance compared to traditional linear approaches.

*LR, GBM, and Decision Tree (DT)*: Graham et al.^28^ analyzed administrative data (120 600 records) from 2 major acute hospitals in Northern Ireland to compare ML algorithms: LR, DT, and GBM for predicting ED admission risk. The GBM outperformed others, achieving an accuracy of 80.31% and an AUROC of 0.859, compared to the DT (accuracy: 80.06%, AUC-ROC: 0.824) and LR (accuracy: 79.94%, AUC-ROC: 0.849).

*RF, DNN, LR, and Gradient Boosted Decision Trees (GBDT)*: De Hond et al^45^ compared ML models and conventional regression techniques for predicting hospitalization of ED patients at 3 time points post-registration. The study utilized data from consecutive ED patients across 3 hospitals in the Netherlands Emergency Department Evaluation Database (NEED). Predictive models for hospitalization were developed using data available at triage, approximately 30 min (including vital signs), and approximately 2 h (including laboratory tests) after ED registration. The models employed ML techniques (RF, GBDT, DNN, and multivariable LR) with covariates including demographics, urgency, presenting complaints, disease severity, and proxies for comorbidity and complexity. Model performance was evaluated using the AUROC curve in independent validation sets from each hospital.

*LR, XGBoost, and DNN*: Hong et al^29^ developed models to predict hospital admission during ED triage by leveraging patient history and triage data. This retrospective study analyzed all adult ED visits resulting in admission or discharge from 1 academic and 2 community EDs between March 2014 and July 2017. They extracted 972 variables per patient visit. The dataset was divided into training (80%), validation (10%), and test (10%) sets. Nine binary classifiers were trained using LR, XGBoost, and DNN across 3 distinct dataset types.

*LR, RF, GBDT, XGBoost, and an ensemble model of the 4 models*: Luo et al^34^ utilized historical data and healthcare professionals’ expertise to create screening rules for automatically prioritizing patient needs. They employed 5 ML methods: LR, RF, GBDT, XGBoost, and an ensemble of these 4 models to sequence and predict outcomes for elective patients. All models demonstrated strong prioritization performance with high predictive values. Notably, XGBoost outperformed others in terms of the AUROC curve, achieving an AUC of 0.901, compared to 0.881 for LR, 0.816 for RF, 0.820 for GBDT, and 0.897 for the ensemble model.

*RF, SVM, and GBT*: Hong et al^30^ developed and assessed an ML approach to predict emergency visits and hospital admissions during radiation and chemoradiation treatments. They analyzed 8134 outpatient radiotherapy (RT) and chemoradiotherapy (CRT) courses from a single institution between 2013 and 2016. Extensive pretreatment data were extracted and processed from the EHR. The dataset was randomly split into training and internal validation cohorts in a 3:1 ratio. GBT, RF, SVM, and Least Absolute Shrinkage and Selection Operator (LASSO) logistic regression models were trained and validated using the AUROC curve. The most predictive model was further evaluated using only disease- and treatment-related features.

*RF and GBC*: Rahimian et al^31^ conducted a study comparing conventional and ML models for predicting first-time emergency admissions. They analyzed longitudinal data from linked EHRs covering 4.6 million patients aged 18 to 100 years, drawn from 389 general practices across England between 1985 and 2015. The dataset was split into a derivation cohort (80%, 3.75 million patients from 300 practices) and a validation cohort (20%, 0.88 million patients from 89 practices), with the cohorts representing geographically distinct regions and varying risk levels. The researchers first replicated a previously established Cox Proportional Hazards (CPH) model to predict the risk of a first emergency admission within 24 months of baseline. They then compared the performance of this model with 2 ML approaches (RF and GBC). Among the models tested, GBC demonstrated the strongest calibration across the full risk spectrum.

*LR, DT, RF, SVM, XGBoost, and Artificial Neural Network (ANN)*: Araz et al^35^ analyzed data from a large metropolitan hospital in the United States that records approximately 50 000 ED visits annually. They applied multiple predictive models, including LR, ANN, DT, RF, SVM, and XGBoost. Model performance was assessed through a series of experiments in which the size of the training and validation datasets varied across multiple years of data. Among the approaches, XGBoost achieved the highest AUC and was also one of the fastest algorithms. Notably, simpler models such as LR also demonstrated strong performance within a reasonable computational timeframe.

*LR, RF, and a random undersampling boosting algorithm*: Fernandes et al^41^ proposed a novel approach to support healthcare professionals in patient triage by stratifying risk and identifying individuals with a higher probability of ICU admission. Their study examined adult patients categorized as Manchester Triage System (MTS) or ESI levels 1 to 3 from EDs in Portugal and the United States. LR, RF, and a random undersampling boosting algorithm were applied. Model performance was compared against a reference model that relied solely on triage priorities, with additional variables incorporated in the experimental models. Across both hospitals, the LR model consistently outperformed the alternatives. In the United States hospital, LR achieved an AUROC of 0.91 (95% CI: 0.90-0.92) and a precision-recall score of 0.30 (95% CI: 0.27-0.33). In the Portugal hospital, the corresponding values were 0.85 (95% CI: 0.83-0.86) and 0.06 (95% CI: 0.05-0.07). Key predictors of ICU admission included heart rate, pulse oximetry, respiratory rate, and systolic blood pressure.

*LR and Neural Network (NN)*: Zhang et al^25^ compared LR and NN models for predicting hospital admission or transfer after initial ED triage, both with and without the integration of natural language processing (NLP) features. Their analysis utilized data from the National Hospital Ambulatory Medical Care Survey (NHAMCS), a cross-sectional probability sample of United States ED visits, specifically from the 2012 and 2013 survey years.

*LR, SVM, Feedforward Neural Network*: El-Bouri et al^42^ introduced an innovative approach for training and regularizing a DL model to predict whether a patient visiting the ED will be admitted to an OUH Trust hospital. This prediction supports timely care and treatment for both the patient and others in the ED. The model achieved AUC scores ranging from 0.60 to 0.78 across different ward types and offered explanations for its predictions, allowing users to prioritize key features for specific wards in future applications.

LR, RF, SVM, and NN: Faisal et al^43^ evaluated LR against other ML methods (RF, SVM, NN) to predict mortality risk in patients after emergency hospital admission, using initial blood test results and physiological measurements. The study employed external validation and analyzed 8937 drug intoxication cases (ICD-10 codes T36.0-T65.9) from 2 149 572 samples in the Korea National Hospital Discharge Survey (KNHDS) spanning 2008 to 2017. Chi-square tests identified factors influencing mortality from drug intoxication, and model performance was compared using IBM SPSS Statistics 25.

*LR, RF, GBDT, DNN, and Lasso Regression*: Raita et al^36^ Raita and Goto 36 utilized ML models to predict clinical outcomes and compared their performance against the conventional ESI. Using data from the NHAMCS ED database (2007-2015), they focused on adult patients (aged ≥18 years). From a 70% randomly sampled training set, they developed 4 ML models (Lasso regression, RF, GBDT, and DNN) using routine triage data (demographics, vital signs, chief complaints, and comorbidities) as predictors. GBDT was built using the R XGBoost package, while DNN employed a 6-layer feedforward model with an adaptive moment estimation optimizer. Hyperparameters, including hidden units, batch size, learning rate, learning rate decay, and dropout rate, were tuned using the R Keras package.

Similarly, Goto et al^15^ investigated ML approaches (Lasso regression, RF, GBDT, and DNN) to predict clinical outcomes and disposition for children in the ED, comparing these with traditional triage methods. This prognostic study analyzed NHAMCS ED data from January 1, 2007, to December 31, 2015, including a nationally representative sample of 52 037 children aged ≤18 years. Data analysis occurred in August 2018. The NHAMCS database represents visits to noninstitutional general and short-stay hospitals across the United States and the District of Columbia, excluding federal, military, and Veterans Affairs hospitals.

*LR, RF, SVM, XGBoost, Light Gradient Boosting Machine (LightGBM), and Multilayer Perceptron (MLP)*: Chen et al^46^ analyzed 52 626 adult ED patients with pneumonia from 3 hospitals between 2010 and 2019. Using 33 feature variables from electronic medical records, they developed an AI model to predict sepsis or septic shock, respiratory failure, and mortality. The study compared predictive accuracies of LR, RF, SVM, LightGBM, MLP, and XGBoost, selecting the best-performing algorithm for each outcome. RF excelled for sepsis or septic shock (AUC = 0.781), while LightGBM performed best for respiratory failure (AUC = 0.847) and mortality (AUC = 0.835). The AI of Things (AIoT)-based model outperformed CURB-65 and the Pneumonia Severity Index (PSI) in predicting mortality (AUC = 0.835 vs 0.681 and 0.835 vs 0.728, respectively).

*LR, DNN, and GTB:* Joseph et al^44^ investigated whether progressively complex DL algorithms could outperform the ESI or vital sign triggers in identifying critically ill patients, using triage data and measured by the AUROC curve. This observational study analyzed a retrospective cohort of adult patients visiting an academic, urban ED at a tertiary care center in the Northeastern United States, with an annual volume of approximately 55 000 visits. All patients from January 1, 2012, to January 1, 2020, were screened. The DL models were developed using TensorFlow, an open-source DL framework.

*Long Short-term Memory (LSTM)*: Kadri et al^37^ developed an LSTM-based DL model to forecast daily ED admissions. The model was tested using experimental data from the pediatric ED at the Lille Regional Hospital Center, France. The results demonstrated the strong potential of the LSTM-based approach for accurately predicting ED admissions.

*LR, DT, KNN, SVM, GBDT, XGBoost, AdaBoost, RF, and MLP*: Elhaj et al^47^ conducted a comparative analysis of 9 supervised ML models to identify the most effective approach for evaluating patient triage outcomes in hospital EDs. The study utilized a retrospective dataset of 2688 patients who visited the ED between April 1, 2020, and June 9, 2020. The dataset included patient demographics (age and gender), vital signs (body temperature, respiratory rate, heart rate, blood pressure, and oxygen saturation), chief complaints, and chronic illness data. Data processing and analysis were performed using Python 3.9 and scientific libraries such as Pandas, NumPy, and Scikit-learn.

*ANN, RF, GLM, NB, and XGBoost*: Liley et al^48^ employed both supervised and unsupervised ML algorithms to develop the SPARRAv4 predictive model. SPARRA*v*4 was applied to routinely collected EHRs from approximately 4.8 million Scottish residents. Using extensive national EHRs from 2013 to 2018, the study focused on creating a model capable of identifying individuals at high risk of emergency admissions. The researchers utilized supervised ML algorithms and incorporated demographic, clinical, and prescription data to enhance predictive accuracy. The model aims to assist healthcare providers in optimizing resource allocation and improving preventative care strategies, ultimately benefiting patient outcomes and system efficiency.

*RF, NN, LR, NB, and NN based on an MLP*: Ricciardi et al^49^ research focused on forecasting ED length of stay (ED-LOS) using ML models. The dataset consisted of 496 172 admissions from 2014 to 2019, representing a hospital in Italy (San Giovanni di Dio e Ruggi d’Aragona University Hospital). This period was chosen to avoid disruptions from the COVID-19 pandemic. Key features included patient gender, age, mode of arrival, triage score, and time of admission, with the outcome variable (ED-LOS) dichotomized into “prolonged stay” (greater than 3 h) or not. These criteria ensured that the dataset remained balanced and relevant for ML model training. Four ML algorithms were employed: RF, NN, LR, Naïve Bayes (NB), and NN based on an MLP. The models were trained using supervised learning techniques, with predictions aimed at classifying patient stays into the defined categories. The models were implemented and tested on the Google Colab platform using Python for development.

*RF, GBM, and LR*: Asmarian et al^50^ research focused on predicting mortality risks among ICU patients who developed infections during their stay, leveraging ML algorithms such as RF, GBM, and LR to analyze patient data effectively. The study utilized a database from the International Nosocomial Infection Control Consortium (INICC), with data prospectively collected from February 2014 to June 2021 across 9 adult medical and surgical ICUs at Nemazee Hospital, Shiraz, Iran. A total of 968 patient records were used for model training and testing, with 317 (32.7%) patients experiencing in-ICU mortality. The models evaluated included RF, gradient boosting machine (GBM), and LR, achieving average AUROC values of 0.77, 0.77, and 0.74, respectively. Sensitivity and specificity for RF, GBM, and LR were (0.65, 0.77), (0.79, 0.62), and (0.74, 0.67), respectively. The Brier scores for RF, GBM, and LR were 0.111, 0.105, and 0.114, respectively.

### ML Tools Used for Data Extraction and Analysis (**RQ2**)

The second research question (**RQ2**) identified the various ML tools used for data extraction, analysis, and optimization in the selected papers. For data extraction, the Structured Query Language (SQL) Server was used.^28,29^ For data analysis, the following tools were used: Keras;^15,36,40^ RStudio^15,25,28-31,34-36,38,40,43,45,48^; TensorFlow^40,44^; Statistical Package for Social Sciences (IBM SPSS)^45^; Matrix Laboratory (MATLAB)^25,26^; Python^31,37,39,40,44,45,47,49^; and Cohen’s Kappa.^41^ Table 6 presents the mapping of each paper to its corresponding category.

**Table 6.** ML Tools Used for Data Extraction, Analysis, and Optimization.

Author	Data extraction	Data analysis					
	SQL	Keras	RStudio	TensorFlow	IBM’s SPSS	MATLAB	Python
26						✓	
38			✓				
39							✓
40		✓	✓	✓			✓
28	✓		✓				
45			✓		✓		✓
29	✓		✓				
34			✓				
30			✓				
31			✓				✓
35			✓				
25			✓			✓	
43			✓				
36		✓	✓				
44				✓			✓
37							✓
47							✓
15		✓	✓				
48			✓				
49							✓

SQL Server is a comprehensive data management system designed to store, process, and safeguard data. It features a programming model based on industry standards and is seamlessly integrated with the Microsoft Distributed Internet Applications (DNA) architecture. It provides various services, such as data extraction, transformation, and loading, specifically tailored to support data warehousing needs.^28,29^

Keras is an application programming interface (API) for implementing neural networks, and it is used for data analysis.^15^ Keras is designed to reduce the cognitive end-user load by shifting the focus away from boilerplate implementation details to the implementation of models. It is a compact and easy-to-learn high-level Python library for DL.^15,36,40^

R-Studio is one of the most used software systems for ML, data mining, and statistics, commonly employed for data analysis.^28^ It supports regression, classification, survival analysis, and clustering with more than 160 modeling techniques.^15,25,28-31,34-36,38,40,43,45,48^ The R-Studio package offers a clean, easy-to-use, and specific language for ML experiments.

TensorFlow is an ML system that operates in heterogeneous and large-scale environments and is used for data analysis.^40,44^ The TensorFlow computational model is based on data flow graphs. It supports a variety of applications, but particularly targets training and inference with DNNs.^59^ An important advantage of using TensorFlow is that the user can employ the graph representation to make many well-defined computations in a single invocation. Since many computations are frequently invoked over a long period, the system may be able to resort to expensive optimizations.^60^

IBM’s SPSS is a software package that is widely used for statistical analysis, data management, and documentation.^45^ It offers a comprehensive suite of statistical and information analysis tools that can be run on a broad range of personal computers.^61^ IBM SPSS is also highly user-friendly, allowing analysts to perform complex analyses without the need for extensive knowledge of the IBM command language.^45^

MATLAB is an interactive programming environment for scientific computing.^26^ It is often used in many technical fields for data analysis, problem-solving, experimentation, and algorithms.^25,26^ More than 60 toolboxes, primarily developed in the MATLAB language, offer enhanced functionality across various specialized technical domains.^26^

Python is a versatile, high-level, object-oriented programming language developed by Guido van Rossum. It has been widely used in recent times.^31,37,39,40,44,45,47,49^ The emphasis on readability in Python’s design allows for clear and concise syntax, enabling programmers to write code more efficiently than in traditional languages such as C.

### Limitations of ML Algorithms Used in EDs (**RQ3**)

**RQ3** investigated the limitations of ML algorithms used in EDs. These limitations are summarized and categorized into DL and data complexity, model accuracy and empirical limitations, single healthcare system data, missing or incomplete data, time-dependent decision-making, generalizability, and limited data in quality registries. Table 7 maps each reviewed paper in relation to each category.

**Table 7.** Limitations of ML Algorithms Used in EDs.

Author	Deep learning and data complexity	Model accuracy and empirical limitations	Single healthcare system data	Missing or incomplete data	Time-dependent decision making	Generalizability	Limited data in quality registries
32		✓					
38		✓					
40				✓			
33			✓				
45						✓	✓
29			✓				
34			✓				
31			✓				
35					✓		
25				✓			
42	✓						

#### DL and Data Complexity

El-Bouri et al^42^ argued that for the DL model to perform better in future investigations, variables such as age, previous admission, and vital signs obtained from the ED, as well as factors more specific to individual ward types, should be included. Additionally, the timing of emergency admissions should be analyzed, and separate models (eg, for males and females) should be developed. Further investigation is needed to determine the most appropriate ward placement for patients during emergencies, based on the specific equipment available in different emergency rooms and the nature of each patient’s condition. However, DL remains a “black box,” as it is difficult to fully understand how the model predicts critical care, although an AI algorithm can be integrated to enhance its accuracy.

#### Model Accuracy and Empirical Limitations

Krämer et al^32^ employed the RF model, which focuses on classification accuracy without considering empirical causality or individual patient visits. Some diagnoses were rarely or never classified. It is significant to note that the classification model (RF) was developed using data that were already available and not related to individual patient visit data. The medical significance of the prediction variables was beyond the scope of the work and thus not considered. The nature of the empirical approach could not provide a classification for primary diagnoses that were never or rarely used. Similarly, the study by Mowbray et al^38^ was limited because the data were not structured to describe the patient’s location after ED registration or the time patients spent in the waiting room. The study was limited by the sample size of 2274, given that ML models perform better with large datasets. Lastly, the data were collected during the daytime, thus limiting the study to daytime admissions only and excluding nighttime admissions.

#### Single Healthcare System Data

Hong et al^29^ study was limited by an under-ascertainment of hospital admissions or emergency visits, given that data from only 1 healthcare system were available. This limitation reduced the ML model’s sensitivity because information on patients who experienced an event at an outside institution was unavailable. In addition, the interpretation of ML was limited due to the complexity of nonlinear and interacting relationships and the presence of correlated factors. Lastly, the potential external accuracy of the model was limited by the structure of the data.

The study conducted by Rahimian et al^31^ divided the EHRs into derivation and validation subsets, which are more prone to model overfitting. Even though this approach was applied, the application of the proposed ML models in other settings may be limited. Therefore, the models proposed by the authors Rahimian et al^31^ require further evaluation. Luo et al^34^ experienced differences between datasets, variables, and models used. They could not compare the performance of the models explicitly with other results presented in the literature review. Additionally, the study sample was limited to 1 dataset from West China Hospital’s ED. Similarly, Yoo et al^33^ experienced the same limitation, as the dataset used was from a single center, and it is unknown how the DeepTriager model performs in other centers. Furthermore, the DeepTriager model must be interactive, and it should be easy for physicians to use.

#### Missing or Incomplete Variables

Some important variables were missing from the literature of the reviewed studies, such as how the patients arrived at the hospital (private transport or by ambulance) and race.^40^ Zhang et al^25^ experienced the same limitation, where missing values in their datasets affected the performance of the predictive models, even though sensitivity analysis was performed to prevent this issue. The extracted information was simplified based on the Bag-of-Words model. However, the model ignored the order of the words, which could lead to specific contextual information being missed. While specific terms were included in the study, they were not classified as having clinical significance in the prediction models.

#### Time-Dependent Decision Making

The study by Araz et al^35^ focused on parameter optimization for SVM and ANN. It was time-consuming, making SVM impractical for real-time decisions in EDs. The study indicated that the procedure takes a significant amount of time (eg, more than 20 min for SVM). Given the specified computational time required for SVM in a classification application, a decision support system using a large number of observations may not find the SVM model practical for time-dependent decisions. This limits its practical use in ED settings.

#### Generalizability

In their study, De Hond et al^45^ pointed out that to construct highly generalizable models, all ED locations need to be employed during the training and testing of the models. This method has the advantage of considering the diversity of sites. However, the pursuit of generalizability may have a negative impact on performance at each specific location. Second, the dependent variable for model training is the clinician’s judgment on patient admission. Moreover, clinical decision-making may be erroneous in and of itself, creating a ceiling effect on the final accuracy of predictive algorithms. Patients’ preferences for hospitalization or their socioeconomic situations could also have an influence. The ceiling effect and the effect of patient preferences, on the other hand, will be equal for traditional regression and ML models, so the key conclusions will remain the same.^45^

#### Limited Data in Quality Registries

A study by De Hond et al^45^ used the NEED, which only comprises variables that are recorded in the hospital information system. As a result, vital signs and blood tests were only provided for those patients whose blood tests and vital signs were taken, limiting model comprehensiveness. Nonetheless, the clinical decision to evaluate these values carries critical prognostic data.^45^

### Recommendations to Address the Limitations (**RQ4**)

The fourth research question (**RQ4**) concerned recommendations to address the limitations. These can be used as a guide for future research, helping to provide solutions to the challenges experienced in EDs. Table 8 highlights these recommendations.

**Table 8.** Recommendations to Address the Limitations.

Author	Expanding data sources and variables	Integration of additional predictors	Algorithm improvements and comparison	Model generalization and clinical implementation
38			✓	
40	✓			
45				✓
29	✓			
34				✓
31			✓	
35		✓		
41		✓		
25	✓			
42	✓			
37		✓		

#### Expanding Data Sources and Variables

Hong et al^29^ suggest that future research should consider expanding the data sources and variables by incorporating additional ones. This implies that variables should be obtained by applying NLP to clinical texts. Additionally, prospective data should identify which acute visits are potentially preventable and which remain a clinical need. Likewise, El-Bouri et al^42^ suggest that further investigations should include additional features to help improve predictions of ward allocation for patients seeking urgent medical attention. Their study aimed to determine which ward best suits each patient at the time of an emergency. Determining this would allow better optimization and allocation of resources (such as beds) for patients requiring emergency admission, enabling them to receive quality care and treatment. In addition, the authors suggest that further studies should include a feature that identifies the ward to which each patient has been allocated when they visit EDs. The ward should have all the required equipment to treat the patient. The authors believe that this will help improve the performance of the models. In addition, Zhang et al^25^ suggest that DL and more complicated structured algorithms should be explored to improve predictive accuracy for ED triage hospital admissions. For large text analysis results, more relevant text mining should be explored using topic models that help identify hidden patterns in the free texts. Identifying the importance of clinical terms by employing a standardized medical lexicon may help improve prediction models. Systems such as biomedical terminology and unified medical language have been exploited and can potentially improve emergency triage predictions. Roquette et al^40^ suggest that future studies focusing on missing but important variables can be implemented to help improve the performance of the models. In addition, symptoms and history of admissions can be used for future work.

#### Integration of Additional Predictors

According to Kadri et al,^37^ future research should test other RNN models, like the Gated Recurrent Unit (GRU), and incorporate information such as pollution peaks, epidemic events, and meteorological data for better patient arrival forecasting at pediatric EDs. Likewise, the proposed future work from Fernandes et al^41^ suggests incorporating the chief complaint as a feature to analyze its impact on model performance. Araz et al^35^ note that in future studies, they will investigate staffing factors for hospital emergency admissions by incorporating staffing-related variables into models and testing the power of predictive models. In addition, to improve the performance of the models, the length of stay (LOS) should be introduced based on a classifier. Finally, simulation models should be introduced for emergency hospital admission predictions.

#### Algorithm Improvements and Comparison

Mowbray et al^38^ suggest that future investigations should focus on improving ML methods to predict other services in health care, such as mortality, LOS, and repeated hospital service usage. Rahimian et al^31^ proposed 2 ML models to predict the risk of patient admissions, and they believe that future studies can be performed to employ additional ML algorithms (eg, RF, SVM, and AI neural networks) to determine the most reliable and efficient models for predicting patient admissions.

#### Model Generalization and Clinical Implementation

De Hond et al^45^ propose that future research should examine whether larger sample sizes or more variables improve the predictive performance of ML models. In addition, future studies should examine the clinical effectiveness of adopting predictive algorithms, as well as the types of situations in which ML models might be preferred over traditional statistical techniques. Moreover, Luo et al^34^ suggest that extending predictive models to other departments or other hospitals could help test their generalizability.

## Discussion

This systematic review shows that ML has been applied across the emergency care continuum using a broad spectrum of algorithms, from LR and RF to gradient-boosting methods, CNNs, recurrent architectures, and LSTM-based time-series models. High-certainty evidence in the GRADE assessment clustered around large national or multicenter ED cohorts evaluating models for core ED decisions such as triage acuity, early admission prediction, critical care, and short-term mortality. This pattern is consistent with recent systematic and scoping reviews, which also find that ML-based triage and admission models generally outperform traditional scores, but that the certainty of evidence is highest when samples are large, outcomes are focused on clinical outcomes, and external validation is reported.^62-66^ Within this context, the present review adds value by systematically mapping not only which algorithms are used in EDs (**RQ1**), but also by grading the certainty of evidence across diverse ED tasks rather than focusing on a single outcome.

A second key observation is that while complex models, such as GBoost, XGBoost, DNN, and LSTM architectures, often deliver the best discrimination in individual studies, simpler approaches, particularly carefully specified LR, frequently remain competitive, especially when predictors are strong and well-curated. This balance between incremental performance gains and model complexity has been described in broader emergency medicine AI overviews and disease-specific triage studies, where ML methods improve risk stratification but sometimes only modestly outperform optimized regression models.^67,68^ At the same time, there is a clear trend toward multi-modal inputs and NLP of triage notes in ED ML research, which can not only capture variations in clinical outcomes but also increase model opacity and implementation burden.^69,70^

The review also provides insights into the data and software ecosystem underpinning ED ML models (**RQ2**). Most of the 27 studies relied on open-source tools such as R, Python, Keras, and TensorFlow alongside SQL-based extraction from EHRs, reflecting the broader move toward flexible, open ML stacks in clinical research and decision support.^71,72^

Clinically and from a policy perspective, the review suggests that ML in EDs is closest to implementation in a few well-defined use cases: risk-based triage support, early admission prediction from triage or early ED data, and targeted risk stratification for specific syndromes such as sepsis, pediatric critical illness, or high-risk respiratory infections. Systematic reviews and empirical evaluations already indicate that AI-enhanced triage and ML-based directives can reduce mis-triage,^73^ support earlier ordering of investigations,^74^ help in managing patient flow^75,76^ and reduce boarding times when embedded into real-time workflows.^77^ At the same time, qualitative and implementation studies stress that successful deployment depends on explainability, calibrated trust, clear role definition for AI versus clinicians, and organizational readiness.^78-80^ The categorization of limitations in this review (**RQ3**), that is, DL “black boxes,” time-dependent decision constraints, registry incompleteness, generalizability issues, and the corresponding recommendations (**RQ4**) directly inform these implementation challenges by indicating where better-designed models, richer data, and prospective impact evaluations are most needed.

This review also advances the field methodologically. Prior reviews have typically focused either on AI in emergency medicine broadly or on a single task such as triage, often without formal evidence grading.^62-64^ In contrast, this work integrates multiple ED tasks (triage, admission, length of stay, forecasting, and ICU outcomes) and applies GRADE to prognostic and prediction-model evidence. This responds to calls for more rigorous, standardized appraisal of AI systems in emergency care and for closer alignment between technical ML performance and evidence-based policy decisions.^81^ Consequently, ML in EDs emerges from this review not just as a set of promising algorithms, but as an uneven evidence base whose maturity varies by task, setting, and design. Future research will need to move beyond model development toward prioritizing external validation,^82^ prospective and controlled trials,^83^ evaluation of equity and fairness,^84^ and integration of explainable AI techniques that support clinician understanding and patient-centered care.^85^

## Conclusion

EDs are primary care entry points where patients’ conditions are assessed to determine what type of care they need and where they should be referred. As such, improving ED processes could help hospitals better manage their resources by providing efficient medical care to those who need it the most. Different studies have proposed models that used different ML or DL algorithms and performance metrics. This indicates that decision-making in EDs can be enhanced by applying the highest-performing models. However, the peer-reviewed studies in this review had limitations, and this paper proposes further research to address these limitations. One suggestion is to conduct a study incorporating additional algorithms and using large datasets to enhance the performance of the ML algorithms. The gaps identified in this systematic review are that most authors mentioned experiencing missing data or additional features that they should have included for their algorithms to perform better, and that the small datasets used reduced the performance of the algorithms. By delineating, among other things, the limitations of the 27 studies included in this review and the means to overcome them, it is anticipated that this review will help researchers improve ED processes using ML in the future.

## Limitations of the Study and Future Research

Like in any SLR, this study has some limitations. Restricting the search to English-language publications, 5 databases, and the years 2017 to 2024 may have introduced selection bias, language bias, and publication bias, potentially excluding relevant studies. As with all SLRs, there is also a risk of attrition bias, selective outcome reporting, and imprecision, which may contribute to Type I or Type II errors when interpreting aggregated findings. This study minimized the potential for these biases by including the quality of evidence assessment using the GRADE framework. To an extent, this quality assessment provided a transparent evaluation of the quality of the selected papers. Limitations also arise from the primary ML methodologies used in the included studies. ML research is prone to sampling bias, class imbalance, algorithmic bias, and overfitting, which can affect model validity and hinder comparability across studies. Variability in datasets, preprocessing techniques, and evaluation metrics across the reviewed papers further adds to this heterogeneity.

Notwithstanding these limitations, the 27 publications reviewed provide valuable insights and a comprehensive analysis of ML applications in EDs based on the research questions posed.

Future studies should broaden database coverage, include non-English publications, and consider a wider publication window. Applying ML-specific appraisal tools such as TRIPOD-ML, PROBAST-AI, and CONSORT-AI may also help assess methodological quality more rigorously and support more rigorous synthesis in future reviews.

## Appendix A

**Table:**

Ref	Year	Description	Risk of bias	Inconsistency	Indirectness	Imprecision	Publication bias	Overall GRADE	Explanation
25	2017	Single system; retrospective ED EHR/text model for admission; internal-only validation.	Serious	Not serious	Serious	Serious	Undetected	Very low	Retrospective single-center development with internal-only validation increases the risk of bias and indirectness. A likely modest sample and limited reporting create serious imprecision, while no conflicting results or explicit publication bias signal were identified.
26	2018	Two EDs; 172 726 visits; e-triage vs ESI for critical care/procedure/admission.	Not serious	Not serious	Not serious	Not serious	Undetected	Moderate	Large multi-site dataset with clear ED outcomes and strong performance supports low risk of bias, directness, and good precision; starting from low (observational), the sample size and robustness justify upgrading to moderate with no domain downgrades.
27	2018	Single health system; ≈6000 ED patients; admission prediction model.	Not serious	Not serious	Not serious	Not serious	Undetected	Low	Methods are sound, but single-system, internal-only validation and a moderate sample size limit generalizability; no domain is seriously compromized, so certainty remains at the observational starting level (low).
28	2018	Two acute hospitals; 120 600 ED records; admission model.	Not serious	Not serious	Not serious	Serious	Undetected	Low	Multi-site data reduce bias and indirectness concerns, but the absence of out-of-system validation and sparse precision reporting justifies serious concern about imprecision; other domains are acceptable, so overall certainty remains low.
29	2018	Large multivariable EHR from one system; admission prediction.	Not serious	Not serious	Not serious	Not serious	Undetected	Moderate	Very large sample and clinically direct ED outcomes with good discrimination minimize risk of bias, inconsistency, indirectness, and imprecision; from a low starting point, this supports upgrading to moderate certainty.
30	2018	Single center; strong reporting (eg, checklist) for admission prediction.	Not serious	Not serious	Not serious	Serious	Undetected	Low	Transparent methods keep the risk of bias low and outcomes direct, but a single-center design and a modest effective sample size create serious imprecision; other domains are not seriously affected, yielding overall low certainty.
31	2018	National/multi-region ED dataset; triage/admission outcomes.	Not serious	Not serious	Not serious	Not serious	Undetected	High	National, multi-region coverage with clinically direct ED outcomes and precise estimates yields no serious issues across any domain; very large, representative data and consistent performance justify upgrading from low to high certainty.
15	2019	Single/dual-site ED; general ML prediction task; internal-only validation.	Serious	Not serious	Serious	Serious	Undetected	Very low	Local, internally validated model with limited reporting and small-to-moderate sample leads to serious concerns about risk of bias, indirectness, and imprecision; no clear inconsistency or publication-bias signal, but cumulative downgrades reduce certainty to very low.
32	2019	Germany; national administrative/DRG data; emergency vs elective and urgency scoring.	Not serious	Not serious	Serious	Not serious	Undetected	Moderate	National scope and large sample provide good precision and low risk of bias, but reliance on administrative classifications to infer ED urgency introduces serious indirectness; overall certainty is upgraded to moderate because national coverage offsets the single-domain limitation.
33	2019	US NHAMCS pediatric national sample; triage predicting critical care/hospitalization.	Not serious	Not serious	Not serious	Not serious	Undetected	High	National pediatric ED sample with directly relevant outcomes and adequate precision shows no serious concerns for bias, inconsistency, indirectness, or imprecision; lack of publication-bias signal and broad representativeness support high-certainty evidence.
34	2019	Single-hospital ED ML study (general outcome); internal validation only.	Serious	Not serious	Serious	Serious	Undetected	Very low	Single-center design, internal-only validation, and narrow catchment create a serious risk of bias and indirectness, while modest sample and limited precision reporting motivate a downgrade for imprecision; with no strong inconsistency or bias signal beyond this, certainty is very low.
35	2019	Single-hospital ED ML model with clear design and internal validation.	Not serious	Not serious	Not serious	Serious	Undetected	Low	Design and reporting are acceptable (no serious bias), and outcomes are direct and consistent, but single-center, internally validated estimates and limited precision justify serious concern about imprecision; certainty therefore remains low.
36	2019	National-level ED dataset; ML vs ESI for critical care/hospitalization.	Not serious	Not serious	Not serious	Not serious	Undetected	High	National coverage, direct clinical outcomes, and robust comparative performance provide precise, generalizable estimates without serious limitations in any domain; this supports upgrading to high certainty.
37	2019	Single pediatric ED site; pediatric outcomes; internal validation.	Serious	Not serious	Serious	Serious	Undetected	Very low	Single-center, pediatric-only context with internal-only validation creates serious concerns regarding bias and indirectness for broader ED practice; relatively modest sample and uncertain event counts further justify serious imprecision, leading to very low certainty.
38	2020	Eight Canadian EDs; 2274 older adults (≥75 years); admission prediction.	Not serious	Not serious	Not serious	Serious	Undetected	Moderate	Multi-site design and standardized assessment produce reasonable risk-of-bias and indirectness profiles, with no evident inconsistency; however, modest sample size and subgroup structure produce serious concerns about imprecision, so overall certainty is moderate.
39	2020	Single tertiary ED; 799 522 visits; early or short-term mortality prediction.	Not serious	Not serious	Not serious	Not serious	Undetected	Moderate	Extremely large within-center dataset and temporal hold-out mitigate bias, inconsistency, and imprecision, but absence of external validation creates some residual indirectness; we therefore avoid upgrading to high and classify overall certainty as moderate.
40	2020	Single pediatric ED; ≈500 k visits; text + DL admission model.	Serious	Not serious	Serious	Not serious	Undetected	Very low	Despite very large N, single pediatric center, internal-only training, and limited interpretability, raise serious risk-of-bias and indirectness concerns for general ED populations; without external validation or cross-setting replication, we treat evidence as very low certainty.
41	2020	Multi-country ED cohort predicting ICU admission/high-acuity outcomes.	Not serious	Not serious	Not serious	Not serious	Undetected	High	Multi-country setting, directly relevant outcomes, adequate sample sizes, and consistent performance leave no serious issues across the 5 domains; from an observational starting point, these strengths justify upgrading to high certainty.
42	2020	Single UK center; outcome is destination/ward-type after ED.	Not serious	Not serious	Serious	Not serious	Undetected	Low	Modeling of ward destination is clinically related but pathway-specific, generating serious indirectness for broader ED decision-making; precision is acceptable, and bias, inconsistency, and publication bias are not major problems, so certainty is low.
43	2020	National Korean ED data with external validation in two hospitals; mortality/admission.	Not serious	Not serious	Not serious	Not serious	Undetected	High	National data plus independent external validation, adequate precision, and direct outcomes reduce concerns in all 5 domains; this combination warrants upgrading the evidence to high certainty.
44	2020	Urban tertiary ED; 445 925 patients; critically ill at triage (ICU≤24 h/mortality).	Not serious	Not serious	Not serious	Not serious	Undetected	High	Very large cohort, clinically direct triage outcomes, and strong discrimination without clear methodological shortcomings yield no serious concerns regarding bias, inconsistency, indirectness, imprecision, or publication bias, supporting high-certainty evidence.
45	2021	Three Dutch EDs (NEED); 172 104 patients; hospitalization at triage (~30 min/~2 h).	Not serious	Not serious	Not serious	Not serious	Undetected	High	Multi-center external validation, assessment of calibration, and large sample sizes minimize risk of bias, indirectness, and imprecision, while consistency across hospitals and absence of clear publication bias justify a high-certainty rating.
46	2021	Taiwan; 3 hospitals; 52 626 ED pneumonia patients; sepsis/respiratory failure/mortality.	Not serious	Not serious	Not serious	Not serious	Undetected	High	Multi-site real-time AIoT implementation, comparison with clinical scores, and adequate precision across outcomes mitigate concerns in all 5 domains, allowing an upgrade to high certainty from a low observational starting point.
47	2023	Single university hospital; 2688 ED patients; multi-class disposition prediction.	Not serious	Not serious	Not serious	Serious	Undetected	Low	Design and outcomes are appropriate and direct, with no strong signal of bias, inconsistency, or publication bias, but the relatively small sample, multi-class structure, and single-center setting lead to serious concerns about imprecision; overall certainty is therefore low.
48	2024	National Scotland; ~4.8 million residents; 12-month emergency admission risk (SPARRA v4).	Not serious	Not serious	Not serious	Not serious	Undetected	High	National population coverage, cross-validation, calibration assessment, and stable subgroup performance provide precise, consistent, and generalizable estimates with no serious limitations across domains, justifying high-certainty evidence.
49	2024	Single Italian ED; 496 172 visits; prolonged ED length-of-stay prediction.	Not serious	Not serious	Serious	Not serious	Undetected	Low	Large sample and acceptable precision limit bias and imprecision concerns, but focus on operational length-of-stay and single-site context creates serious indirectness for broader clinical outcomes, keeping overall certainty at a low level.
50	2024	Nine ICUs (Iran); 968 patients with device-associated infections; ICU mortality.	Not serious	Not serious	Serious	Not serious	Undetected	Low	Multi-ICUs setting improves representativeness, but the ICU context and infection-focused cohort mean serious indirectness for ED populations; sample size and results are reasonably precise, and no major bias or inconsistency is evident, yielding low overall certainty.

## References

[1] Rasouli HR Aliakbar Esfahani A Abbasi Farajzadeh M. Challenges, consequences, and lessons for way–outs to emergencies at hospitals: a systematic review study. BMC Emerg Med. 2019;19:1-10. 31666023 10.1186/s12873-019-0275-9 PMC6822347

[2] do Nascimento Rocha HM da Costa Farre AGM de Santana Filho VJ. Adverse events in emergency department boarding: a systematic review. J Nurs Scholarsh. 2021;53:458-467. 33792131 10.1111/jnu.12653

[3] Darraj A Hudays A Hazazi A , et al. The association between emergency department overcrowding and delay in treatment: a systematic review. Healthcare. 2023;11(3):385. 36766963 10.3390/healthcare11030385 PMC9914164

[4] National Center for Health Statistics. Emergency department visit rates by selected characteristics: United States, 2021. 2023. Accessed September 25, 2025. https://www.cdc.gov/nchs/data/databriefs/db478.pdf 37642973

[5] Jarvis PRE . Improving emergency department patient flow. Clin Exp Emerg Med. 2016;3:63. 27752619 10.15441/ceem.16.127 PMC5051606

[6] American Nurses Association. Third Edition. Principles for nurse staffing. 2019. Accessed September 25, 2025. https://www.nursingworld.org/practice-policy/nurse-staffing/staffing-principles/

[7] Ahsan KB Alam M Morel DG , et al. Emergency department resource optimisation for improved performance: a review. J Ind Eng Int. 2019;15:253-266.

[8] National Academies of Sciences, Engineering, and Medicine. Integrating Social Care into the Delivery of Health Care: Moving Upstream to Improve the Nation’s Health. National Academies Press; 2019. Accessed October 15, 2025. https://www.ncbi.nlm.nih.gov/books/NBK552597/doi: 10.17226/25467 31940159

[9] Tschoellitsch T Seidl P Böck C , et al. Using emergency department triage for machine learning-based admission and mortality prediction. Eur J Emerg Med. 2023;30:408-416. 37578440 10.1097/MEJ.0000000000001068

[10] Lee J-T Hsieh C-C Lin C-H , et al. Prediction of hospitalization using artificial intelligence for urgent patients in the emergency department. Sci Rep. 2021;11:19472. 34593930 10.1038/s41598-021-98961-2 PMC8484275

[11] Janke AT Overbeek DL Kocher KE , et al. Exploring the potential of predictive analytics and big data in emergency care. Ann Emerg Med. 2016;67:227-236. 26215667 10.1016/j.annemergmed.2015.06.024

[12] Vela VJ Rontala SP Oluwatoyin OL. Facilitating conditions in adopting big data analytics at medical aid organizations in South Africa. J Ind Distrib Bus. 2022;13:1-10.

[13] Harrou F Dairi A Kadri F , et al. Effective forecasting of key features in hospital emergency department: hybrid deep learning-driven methods. Mach Learn Appl. 2022;7:100200.

[14] Rasool S Husnain A Saeed A , et al. Harnessing predictive power: exploring the crucial role of machine learning in early disease detection. Jurnal Inovasi dan Humaniora. 2023;1:302-315.

[15] Goto T Camargo CA Faridi MK , et al. Machine learning–based prediction of clinical outcomes for children during emergency department triage. JAMA Netw Open. 2019;2:e186937. 10.1001/jamanetworkopen.2018.6937 PMC6484561 30646206

[16] Taylor RA Pare JR Venkatesh AK , et al. Prediction of in-hospital mortality in emergency department patients with sepsis: a local big data–driven, machine learning approach. Acad Emerg Med. 2016;23:269-278. 26679719 10.1111/acem.12876 PMC5884101

[17] Williams Jr RI Clark LA Clark WR , et al. Re-examining systematic literature review in management research: additional benefits and execution protocols. Eur Manag J. 2021;39:521-533.

[18] Sarkis-Onofre R Catalá-López F Aromataris E , et al. How to properly use the PRISMA Statement. Syst Rev. 2021;10:1-3. 33875004 10.1186/s13643-021-01671-z PMC8056687

[19] García-Peñalvo FJ. Developing robust state-of-the-art reports: systematic literature reviews. Educ Knowl Soc. 2022;23:e28600.

[20] Hiebl MR. Sample selection in systematic literature reviews of management research. Organ Res Methods. 2023;26:229-261.

[21] Page MJ McKenzie JE Bossuyt PM , et al. The PRISMA 2020 statement: an updated guideline for reporting systematic reviews. BMJ. 2021;372:n71. 10.1136/bmj.n71 PMC8005924 33782057

[22] Rahmadhan P Wana MA Sensuse DI , et al. Trends and applications of gamification in e-commerce: a systematic literature review. J Inf Syst Eng Bus Intell. 2023;9:28-37.

[23] Mory-Alvarado A Juiz C Bermejo B , et al. Green IT in small and medium-sized enterprises: a systematic literature review. Sustain Comput Inform Syst. 2023;39:100891.

[24] Maharani NZ Kurniawan SS Sensuse DI , et al. Motivations and potential solutions in developing a knowledge management system for organization at higher education: a systematic literature review. J Inf Syst Eng Bus Intell. 2024;10:270-289.

[25] Zhang X Kim J Patzer RE , et al. Prediction of emergency department hospital admission based on natural language processing and neural networks. Methods Inf Med. 2017;56:377-389. 28816338 10.3414/ME17-01-0024

[26] Levin S Toerper M Hamrock E , et al. Machine-learning-based electronic triage more accurately differentiates patients with respect to clinical outcomes compared with the emergency severity index. Ann Emerg Med. 2018;71:565-574.e562. 10.1016/j.annemergmed.2017.08.005 28888332

[27] Qiao Z Sun N Li X , et al. Using machine learning approaches for emergency room visit prediction based on electronic health record data. Stud Health Technol Inform. 2018;247:111-115. 29677933

[28] Graham B Bond R Quinn M , et al. Using data mining to predict hospital admissions from the emergency department. IEEE Access. 2018;6:10458-10469.

[29] Hong WS Haimovich AD Taylor RA. Predicting hospital admission at emergency department triage using machine learning. PLoS ONE. 2018;13:e0201016. 10.1371/journal.pone.0201016 PMC6054406 30028888

[30] Hong JC Niedzwiecki D Palta M , et al. Predicting emergency visits and hospital admissions during radiation and chemoradiation: an internally validated pretreatment machine learning algorithm. JCO Clin Cancer Inform. 2018;2:1-11. 10.1200/CCI.18.00037 30652595

[31] Rahimian F Salimi-Khorshidi G Payberah AH , et al. Predicting the risk of emergency admission with machine learning: development and validation using linked electronic health records. PLoS Med. 2018;15:e1002695. 10.1371/journal.pmed.1002695 PMC6245681 30458006

[32] Krämer J Schreyögg J Busse R. Classification of hospital admissions into emergency and elective care: a machine learning approach. Health Care Manag Sci. 2019;22:85-105. 29177993 10.1007/s10729-017-9423-5

[33] Yoo I Bi J Hu X. National Science Foundation (US), and Institute of Electrical and Electronics Engineers. Paper presented at: Proceedings, 2019 IEEE International Conference on Bioinformatics and Biomedicine; November 18-21, 2019; San Diego, CA, USA.

[34] Luo L Li J Liu C , et al. Using machine-learning methods to support health-care professionals in making admission decisions. Int J Health Plann Manage. 2019;34:e1236-e1246. 10.1002/hpm.2769 30957270

[35] Araz OM Olson D Ramirez-Nafarrate A. Predictive analytics for hospital admissions from the emergency department using triage information. Int J Prod Econ. 2019;208:199-207.

[36] Raita Y Goto T Faridi MK , et al. Emergency department triage prediction of clinical outcomes using machine learning models. Crit Care. 2019;23:1-13. 30795786 10.1186/s13054-019-2351-7 PMC6387562

[37] Kadri F Baraoui M Nouaouri I. An LSTM-based deep learning approach with application to predicting hospital emergency department admissions. Paper presented at: 2019 International Conference on Industrial Engineering and Systems Management (IESM) 2019; September 25-29, 2019; Shanghai, China; 2019:1-6.

[38] Mowbray F Zargoush M Jones A , et al. Predicting hospital admission for older emergency department patients: insights from machine learning. Int J Med Inform. 2020;140:104163. 32474393 10.1016/j.ijmedinf.2020.104163

[39] Klug M Barash Y Bechler S , et al. A gradient boosting machine learning model for predicting early mortality in the emergency department triage: devising a nine-point triage score. J Gen Intern Med. 2020;35:220-227. 31677104 10.1007/s11606-019-05512-7 PMC6957629

[40] Roquette BP Nagano H Marujo EC , et al. Prediction of admission in pediatric emergency department with deep neural networks and triage textual data. Neural Netw. 2020;126:170-177. 32240912 10.1016/j.neunet.2020.03.012

[41] Fernandes M Mendes R Vieira SM , et al. Predicting intensive care unit admission among patients presenting to the emergency department using machine learning and natural language processing. PLoS ONE. 2020;15:e0229331. 10.1371/journal.pone.0229331 PMC7053743 32126097

[42] El-Bouri R Eyre DW Watkinson P , et al. Hospital admission location prediction via deep interpretable networks for the year-round improvement of emergency patient care. IEEE J Biomed Health Inform. 2020;25:289-300. 10.1109/JBHI.2020.2990309 32750898

[43] Faisal M Scally A Howes R , et al. A comparison of logistic regression models with alternative machine learning methods to predict the risk of in-hospital mortality in emergency medical admissions via external validation. Health Inform J. 2020;26:34-44. 10.1177/1460458218813600 30488755

[44] Joseph JW Leventhal EL Grossestreuer AV , et al. Deep-learning approaches to identify critically Ill patients at emergency department triage using limited information. J Am Coll Emerg Physicians Open. 2020;1:773-781. 33145518 10.1002/emp2.12218 PMC7593422

[45] De Hond A Raven W Schinkelshoek L , et al. Machine learning for developing a prediction model of hospital admission of emergency department patients: hype or hope? Int J Med Inform. 2021;152:104496. 34020171 10.1016/j.ijmedinf.2021.104496

[46] Chen YM Kao Y Hsu CC , et al. Real-time interactive artificial intelligence of things–based prediction for adverse outcomes in adult patients with pneumonia in the emergency department. Acad Emerg Med. 2021;28:1277-1285. 34324759 10.1111/acem.14339

[47] Elhaj H Achour N Tania MH , et al. A comparative study of supervised machine learning approaches to predict patient triage outcomes in hospital emergency departments. Array. 2023;17:100281.

[48] Liley J Bohner G Emerson SR , et al. Development and assessment of a machine learning tool for predicting emergency admission in Scotland. NPJ Digit Med. 2024;7:277. 39443624 10.1038/s41746-024-01250-1 PMC11499905

[49] Ricciardi C Marino MR Trunfio TA , et al. Evaluation of different machine learning algorithms for predicting the length of stay in the emergency departments: a single-centre study. Front Digit Health. 2024;5:1323849. 38259256 10.3389/fdgth.2023.1323849 PMC10800466

[50] Asmarian N Zand F Sabetian G , et al. Machine learning-derived analysis of intensive care unit mortality in patients with ICU acquired infections. J Crit Care. 2024;81:154746.

[51] Mallett R Hagen-Zanker J Slater R , et al. The benefits and challenges of using systematic reviews in international development research. J Dev Effect. 2012;4:445-455.

[52] Guyatt G Oxman AD Akl EA , et al. GRADE guidelines: 1. Introduction—GRADE evidence profiles and summary of findings tables. J Clin Epidemiol. 2011;64:383-394. 21195583 10.1016/j.jclinepi.2010.04.026

[53] Iorio A Spencer FA Falavigna M , et al. Use of GRADE for assessment of evidence about prognosis: rating confidence in estimates of event rates in broad categories of patients. BMJ. 2015;350:h870. doi:10.1136/bmj.h870 25775931

[54] Huguet A Hayden JA Stinson J , et al. Judging the quality of evidence in reviews of prognostic factor research: adapting the GRADE framework. Syst Rev. 2013;2:71. 24007720 10.1186/2046-4053-2-71 PMC3930077

[55] Foroutan F Guyatt G Zuk V , et al. GRADE guidelines 28: use of GRADE for the assessment of evidence about prognostic factors: rating certainty in identification of groups of patients with different absolute risks. J Clin Epidemiol. 2020;121:62-70. 31982539 10.1016/j.jclinepi.2019.12.023

[56] Balshem H Helfand M Schünemann HJ , et al. GRADE guidelines: 3. Rating the quality of evidence. J Clin Epidemiol. 2011;64:401-406. 21208779 10.1016/j.jclinepi.2010.07.015

[57] Brozek JL Canelo-Aybar C Akl EA , et al. GRADE guidelines 30: the GRADE approach to assessing the certainty of modeled evidence—an overview in the context of health decision-making. J Clin Epidemiol. 2021;129:138-150. 32980429 10.1016/j.jclinepi.2020.09.018 PMC8514123

[58] Schünemann HJ Higgins JP Vist GE , et al. Completing ‘Summary of findings’ tables and grading the certainty of the evidence. In: Higgins JPT Thomas J Chandler J , et al., eds. Cochrane Handbook for Systematic Reviews of Interventions. John Wiley & Sons; 2019:375-402.

[59] Abadi M. TensorFlow: learning functions at scale. Paper presented at: Proceedings of the 21st ACM SIGPLAN International Conference on Functional Programming; September 18-24, 2016; Nara, Japan.

[60] Abadi M Isard M Murray DG. A computational model for TensorFlow: an introduction. Paper presented at: Proceedings of the 1st ACM SIGPLAN International Workshop on Machine Learning and Programming Languages, Barcelona, Spain, June 18, 2017:1-7.

[61] Obertová Z Stewart A Cattaneo C. Statistics and Probability in Forensic Anthropology. Academic Press; 2020.

[62] Almulihi QA Alquraini AA Almulihi FAA , et al. Applications of artificial intelligence and machine learning in emergency medicine triage – a systematic review. Med Arch. 2024;78:198. 39944197 10.5455/medarh.2024.78.198-206 PMC11813208

[63] Da’Costa A Teke J Origbo JE , et al. AI-driven triage in emergency departments: a review of benefits, challenges, and future directions. Int J Med Inform. 2025;197:105838. 39965433 10.1016/j.ijmedinf.2025.105838

[64] Tahernejad A Sahebi A Abadi ASS , et al. Application of artificial intelligence in triage in emergencies and disasters: a systematic review. BMC Public Health. 2024;24:3203. 39558305 10.1186/s12889-024-20447-3 PMC11575424

[65] Gao F Boukebous B Pozzar M , et al. Predictive models for emergency department triage using machine learning: a systematic review. Obstet Gynecol Res. 2022;5:136-157.

[66] Mueller B Kinoshita T Peebles A , et al. Artificial intelligence and machine learning in emergency medicine: a narrative review. Acute Med Surg. 2022;9:e740. 10.1002/ams2.740 PMC8887797 35251669

[67] Fenn A Davis C Buckland DM , et al. Development and validation of machine learning models to predict admission from emergency department to inpatient and intensive care units. Ann Emerg Med. 2021;78:290-302. 33972128 10.1016/j.annemergmed.2021.02.029

[68] Chiu YM Courteau J Dufour I , et al. Machine learning to improve frequent emergency department use prediction: a retrospective cohort study. Sci Rep. 2023;13:1981. 36737625 10.1038/s41598-023-27568-6 PMC9898278

[69] Chang Y-H Lin Y-C Huang F-W , et al. Using machine learning and natural language processing in triage for prediction of clinical disposition in the emergency department. BMC Emerg Med. 2024;24:237. 39695961 10.1186/s12873-024-01152-1 PMC11657801

[70] Stewart J Lu J Goudie A , et al. Applications of natural language processing at emergency department triage: a narrative review. PLoS ONE. 2023;18:e0279953. 10.1371/journal.pone.0279953 PMC10721204 38096321

[71] Adamson B Waskom M Blarre A , et al. Approach to machine learning for extraction of real-world data variables from electronic health records. Front Pharmacol. 2023;14:1180962. 37781703 10.3389/fphar.2023.1180962 PMC10541019

[72] Carbonaro A Giorgetti L Ridolfi L , et al. From raw data to research-ready: a FHIR-based transformation pipeline in a real-world oncology setting. Comput Biol Med. 2025;197:111051. 40945218 10.1016/j.compbiomed.2025.111051

[73] Abdalhalim AZA Ahmed SNN Ezzelarab AMD , et al. Clinical impact of artificial intelligence-based triage systems in emergency departments: a systematic review. Cureus. 2025;17:e85667. 10.7759/cureus.85667 PMC12241827 40642667

[74] Singh D Nagaraj S Mashouri P , et al. Assessment of machine learning–based medical directives to expedite care in pediatric emergency medicine. JAMA Netw Open. 2022;5:e222599. 10.1001/jamanetworkopen.2022.2599 PMC8928004 35294539

[75] Leonard F Gilligan J Barrett MJ. Development of a low-dimensional model to predict admissions from triage at a pediatric emergency department. JACEP Open. 2022;3:e12779. 10.1002/emp2.12779 PMC9286530 35859857

[76] Taylor RA Chmura C Hinson J , et al. Impact of artificial intelligence–based triage decision support on emergency department care. NEJM AI. 2025;2:AIoa2400296.

[77] Kim E Han KS Cheong T , et al. Analysis on benefits and costs of machine learning-based early hospitalization prediction. IEEE Access. 2022;10:32479-32493.

[78] Liu Y Liu C Zheng J , et al. Improving explainability and integrability of medical AI to promote health care professional acceptance and use: mixed systematic review. J Med Internet Res. 2025;27:e73374. 10.2196/73374 PMC12371287 40773743

[79] Huguet N Chen J Parikh RB , et al. Applying machine learning techniques to implementation science. Online J Public Health Inform. 2024;16:e50201. 10.2196/50201 PMC11074902 38648094

[80] Darvish M Holst J-H Bick M. Explainable AI in healthcare: factors influencing medical practitioners’ trust calibration in collaborative tasks. Paper presented at: Proceedings of the 57th Hawaii International Conference on System Sciences; January 3-6, 2024; Hawaii, USA Honolulu: AIS Electronic Library; 2024:3326-3335. Tung X. Bui.

[81] Chenais G Lagarde E Gil-Jardiné C. Artificial intelligence in emergency medicine: viewpoint of current applications and foreseeable opportunities and challenges. J Med Internet Res. 2023;25:e40031. 10.2196/40031 PMC10245226 36972306

[82] Jhaveri A Abolhassani F Fine B. Prospective external validation of an AI-based emergency department pneumonia disposition prediction tool. Can Assoc Radiol J. 2025;76:664-673. 40008968 10.1177/08465371251320938

[83] Kirubarajan A Taher A Khan S , et al. Artificial intelligence in emergency medicine: a scoping review. JACEP Open. 2020;1:1691-1702. 33392578 10.1002/emp2.12277 PMC7771825

[84] Liu M Ning Y Teixayavong S , et al. A scoping review and evidence gap analysis of clinical AI fairness. NPJ Digit Med. 2025;8:360. 40517148 10.1038/s41746-025-01667-2 PMC12167363

[85] Johannssen A Chukhrova N. The crucial role of explainable artificial intelligence (XAI) in improving health care management. Health Care Manag Sci. 2025;28(3):565-570. 41026402 10.1007/s10729-025-09720-y PMC12535480

